# Endothelial glycocalyx remodeling and galectin signaling in aging associated epilepsy

**DOI:** 10.3389/fneur.2026.1961370

**Published:** 2026-10-19

**Authors:** Adam Legradi, Cristiana Pareo, Mohd Yaqub Mir

**Affiliations:** 1 Department of Cell Biology and Molecular Medicine, University of Szeged, Szeged, Hungary; 2 Department of Neuroscience and Rehabilitation, University of Ferrara, Ferrara, Italy; 3 Epilepsy Centre, Department of Clinical Sciences, Lund University Hospital, Lund, Sweden

**Keywords:** blood-brain barrier, brain aging, endothelial glycocalyx, epileptogenesis, galectin-1, galectin-3, neuroinflammation

## Abstract

Physiological aging can alter the brain endothelial glycocalyx and blood–brain barrier (BBB), while experimental seizures independently engage galectin-3 (Gal-3) and inflammasome-associated inflammatory signaling. This narrative review critically examines whether these observations justify a shared mechanism in aging-associated epilepsy. Primary studies support endothelial mucin-type O-glycosylation as a determinant of BBB integrity and Gal-3 as a modulator of inflammation in selected seizure models. They do not establish that endothelial glycan remodeling changes Gal-3 binding or initiates epileptogenesis. We distinguish extracellular galectin-glycan recognition from intracellular inflammasome signaling and address the anatomical separation between parenchymal microglia and the luminal glycocalyx. Glycan elongation, ligand density, terminal modifications, and steric accessibility could alter binding in different directions; uniform exposure of galectin ligands cannot be assumed. Galectin-1 also has context-dependent protective and detrimental effects. We propose compartment-resolved experiments combining glycan measurements, cell-specific perturbations, BBB assessment, and longitudinal electroencephalography to test the convergence hypothesis.

## 1 Introduction

The neurovascular unit includes endothelial cells, pericytes, astrocytes, microglia, neurons, and extracellular matrix components. Its cellular interactions regulate exchange between blood and brain and help maintain an environment compatible with neuronal signaling (1, 2). BBB dysfunction in physiological aging must be distinguished from vascular injury associated with Alzheimer’s disease, stroke, and other conditions that become more frequent in older people (3–5).

The endothelial glycocalyx is a carbohydrate-rich surface layer involved in vascular permeability, mechanotransduction, and cell interactions (6, 7). Recent mouse experiments establish a contribution of endothelial mucin-type O-glycosylation to BBB integrity during aging (8). Separately, experiments in seizure models implicate Gal-3 in microglial inflammatory responses (9). These findings motivate a question rather than establish a mechanism: does aging-related endothelial glycan remodeling alter galectin engagement in a compartment that influences seizure susceptibility or the development of epilepsy?

This review first describes the clinical and cellular background, then examines endothelial glycosylation and galectin experiments in their original systems. The convergence hypothesis is introduced only after these evidence streams have been evaluated. Throughout, a demonstrated result refers to the measured outcome in a specified model; a mechanistic precedent refers to an observation in another biological setting; and a hypothesis refers to a relationship that has not been directly tested in the aging epileptic brain.

### 1.1 Review approach and evidence boundaries

This is a hypothesis-driven narrative review, not a systematic review or meta-analysis. Its focus is the critical interpretation of selected literature on endothelial glycocalyx structure, mucin-type O-glycosylation, galectins, BBB dysfunction, aging, and experimental epilepsy. Primary studies support experimental claims; reviews provide broader context. Because the proposed causal bridge has not been demonstrated, the discussion evaluates the model, intervention, measured endpoint, and limitation of each central study instead of combining independent observations into an established pathway.

Physiological aging evidence is considered separately from neurodegenerative disease evidence. An induced status epilepticus model is also distinguished from chronic epilepsy: acute behavioral seizures, tissue injury, or cytokine changes do not alone demonstrate persistent spontaneous recurrent seizures or modification of epileptogenesis. Findings in cultured microglia, astrocytes, or endothelial cell lines resolve particular cellular processes but cannot reproduce the full neurovascular architecture or aging phenotype.

## 2 Epilepsy and aging

Epileptic seizures reflect abnormal excessive or synchronous neuronal activity; epilepsy involves an enduring predisposition to generate seizures (10). Epileptogenesis describes the development of that persistent predisposition. These terms identify different outcomes, and an intervention that reduces seizure severity or post-seizure injury is not necessarily an intervention that prevents epilepsy. Late-onset epilepsy is often discussed as epilepsy beginning around or after 60 years of age, although age thresholds vary between studies (11).

The Rotterdam Study provides epidemiological context, not evidence for a glycan mechanism. Its population-based door-to-door assessment in Ommoord, Rotterdam, during 1991–1993 included 5,559 people aged 55–95 years. Overall active epilepsy prevalence was 0.9% when special syndromes were included and 0.8% when they were excluded; prevalence increased with age (12). This was a prevalence study, so it neither identifies a molecular cause nor demonstrates that physiological aging alone causes epilepsy.

Cerebrovascular disease, neurodegenerative disorders, tumors, and previous brain injury account for substantial seizure risk in older populations (11). Alzheimer-associated seizure observations therefore cannot be used as direct evidence of a mechanism in uncomplicated aging (13). For unexplained late-onset epilepsy, age-dependent vascular or inflammatory vulnerability remains a possible contributor that requires direct investigation.

## 3 Inflammation and the cellular environment of seizures

Neurons generate seizure activity, while astrocytes regulate extracellular potassium and glutamate and contribute to metabolic support. Microglia coordinate innate immune responses, and endothelial cells and pericytes regulate vascular exchange. Seizures can change these supporting cell states; inflammatory mediators can in turn affect neuronal excitability and vascular function. Experimental and human temporal lobe epilepsy studies document complement-related responses, while kainate experiments demonstrate time-dependent hippocampal eicosanoid production (14, 15). These observations support multiple inflammatory processes rather than a single pathway shared by every epilepsy.

Pilocarpine is a muscarinic agonist used to induce prolonged seizures, often after lithium sensitization. Kainic acid is an excitatory glutamate-receptor agonist used in seizure models and cell-stimulation experiments. A kainate-exposed glial culture models a chemical inflammatory or injury response; it does not itself have epileptic network activity. This distinction is particularly important when interpreting the Gal-3 and NLRP3 studies discussed below.

Vascular inflammation is also experimentally tractable. Fabene et al. (16) investigated leukocyte-endothelial adhesion in a mouse seizure model and found that interfering with adhesion reduced seizure-related pathology. Librizzi et al. (17) demonstrated induction of endothelial adhesion factors during epileptiform activity in an isolated guinea pig brain preparation *in vitro*. Neither experiment measured the proposed aging-dependent endothelial galectin-binding change. Moreover, leukocyte recruitment should not be assumed to accompany all forms of aging-related BBB dysfunction.

## 4 BBB dysfunction and astrocytic mechanisms

The BBB maintains a selective interface through endothelial junctions, low nonspecific transport, and interactions with surrounding cells (1). When blood-derived proteins enter brain tissue, they can alter the extracellular environment and glial signaling. Albumin extravasation is therefore relevant because it offers a defined connection between vascular leakage and network dysfunction, rather than simply being a marker of barrier injury.

Ivens et al. (18) used focal BBB disruption or direct albumin exposure in rats, together with albumin-exposed brain slices *in vitro*. They observed astrocytic albumin uptake associated with TGF-beta receptor signaling, reduced expression of the inward-rectifying potassium channel Kir4.1, and impaired potassium buffering. This supplies experimental support for a BBB-astrocyte route to epileptiform changes. Astrocytic glutamate transport is another determinant of extracellular homeostasis, but the broader glutamate-transporter literature should not be presented as evidence that Gal-3 specifically impairs transport through the proposed endothelial mechanism (19).

### 4.1 Evidence from aging mice and human imaging

Evidence for ordinary aging is distinct. Elahy et al. compared healthy female C57BL/6 J mice at 3 and 24 months, with six animals per group. Confocal assessment of parenchymal IgG and endothelial flow cytometry showed greater leakage and reduced tight-junction protein expression in aged animals. Increased endothelial TNF-alpha was associated with these abnormalities; increased adhesion molecule expression and leukocyte recruitment were not demonstrated (20). Association in this aging comparison does not establish that TNF-alpha alone caused the phenotype.

In humans, Montagne et al. (21) used dynamic contrast-enhanced MRI to assess BBB permeability. The cognitively unimpaired group included 24 individuals aged 23–91 years, and hippocampal permeability increased with age. These imaging data support region-specific vascular aging but do not resolve glycocalyx composition, galectin engagement, or subsequent epilepsy. Rodents aged approximately 18–24 months are commonly used to study late-life biology; their phenotypes cannot be translated to a fixed human age because vascular disease, lifespan, and immune history differ between species.

### 4.2 Transport selectivity and regional heterogeneity during aging

BBB aging is not a uniform increase in permeability to every blood constituent. Yang et al. (22) compared male C57BL/6 mice at 3 and 20–24 months using labeled plasma proteins, transport ligands, endothelial profiling, and vesicle measurements. Uptake of plasma depleted of albumin and IgG decreased in aged brains, whereas IgG accumulation increased. Their results supported a shift from receptor-mediated transport toward nonspecific caveolar transcytosis, alongside reduced pericyte coverage. Thus, declining physiological protein delivery can coexist with increased entry of selected macromolecules. A single tracer does not capture all transport changes, and these findings do not establish Gal-3 transport or glycocalyx-dependent galectin signaling.

Regional differences further qualify the concept of barrier aging. Bravo-Ferrer et al. examined C57BL/6 J mice of both sexes at 2–3 and 18–20 months using immunofluorescence, electron microscopy, and permeability assays across the prefrontal cortex, hippocampus, and corpus callosum. Changes were subtle and region-dependent; the prefrontal cortex showed the greatest combination of endothelial, permeability, basement-membrane, and astrocyte-endfoot alterations (23). These results caution against extrapolating a hippocampal measurement to the whole brain. Differences in age, region, tracer, and transport route should therefore be considered when comparing aging studies or selecting regions in which to test the convergence hypothesis.

## 5 Endothelial glycocalyx composition and aging experiments

### 5.1 Glycan structures before functional interpretation

The endothelial glycocalyx contains glycoproteins, proteoglycans bearing glycosaminoglycans, and glycolipids. Heparan sulfate, chondroitin sulfate, and hyaluronan contribute to its hydrated architecture, while N-linked and O-linked glycans decorate distinct protein scaffolds (6, 24, 25). Thickness, density, molecular composition, and turnover are separate properties. A thinner layer does not establish that every component has decreased or that its remaining glycans bind more galectin.

Mucin-type O-glycosylation begins with GalNAc attachment to serine or threonine, generating the Tn structure. C1GALT1 adds galactose to form core 1, Gal-beta1-3-GalNAc-alpha-O-Ser/Thr; further extension and branching can generate more complex structures (8, 26, 27). Core 1 should not be equated with the LacNAc motif, Gal-beta1-4-GlcNAc. LacNAc-containing ligands can occur on extended glycans, N-glycans, and glycolipids. Thus, reduced core 1 synthesis could remove or change some ligands rather than necessarily expose or increase them.

### 5.2 Direct experimental evidence in aging brain endothelium

Shi et al. studied male C57BL/6 mice, including 3- versus 21-month structural comparisons and 3- versus 19-month transcriptomic data. Lanthanum-enhanced electron microscopy and glycan-selective profiling showed reduced glycocalyx thickness and mucin-domain labeling, increased glycosaminoglycan signals, and no significant change in the measured alpha2,3- or alpha2,6-sialic-acid signals. Endothelial C1galt1 knockdown in young mice increased tracer, albumin, and IgG leakage. In bEnd.3 endothelial cells, glycosyltransferase manipulation altered mucin labeling; mucinase treatment increased oxidative stress. The catalytically inactive StcE(E447D) probe measured mucin-domain labeling, whereas active StcE disrupted mucin domains (8).

For rescue experiments, endothelial-targeted AAV constructs were administered at 17 months, with BBB assessment 8 weeks later. Both C1GALT1 and B3GNT3 restoration improved labeling and reduced tracer leakage. Cognitive benefits in Y-maze and contextual fear-conditioning tests at 12 weeks were significant for B3GNT3, not C1GALT1; inflammatory benefits also differed between constructs. These interventions support a contribution of endothelial glycosylation to BBB homeostasis, but galectin binding and seizures were not tested. The male-only design limits inference about sex effects (8).

### 5.3 Biophysical precedent outside the aging brain

Shurer et al. (28) manipulated mucin-rich cell surfaces, including engineered MCF10A epithelial cells and carcinoma cell lines, and examined membrane morphology. Changing mucin abundance and digesting mucins altered surface protrusions; a polymer-brush model related polymer size and density to membrane curvature. This demonstrates that glycocalyx polymers can generate physical effects at membranes. It does not identify an aging endothelial Gal-3 ligand or show that thinner glycocalyx increases galectin signaling.

Paszek et al. (29) combined synthetic glycopolymer mimetics and MUC1 expression in mammary epithelial systems with imaging and adhesion assays. Bulky surface polymers affected integrin organization, focal adhesions, and growth-related signaling. This is a specific example of glycocalyx-dependent receptor organization in a non-neural system. Its direction of effect cannot simply be reversed to predict that age-related mucin reduction activates endothelial receptors. The cancer glycocalyx literature supplies physical precedent, while the required brain endothelial binding experiment remains absent (30).

## 6 Galectin recognition and candidate structural changes

Galectins recognize beta-galactoside-containing glycans, with affinity dependent on the complete glycan structure and its presentation (31, 32). Prototype galectins such as Gal-1 have one carbohydrate-recognition domain per polypeptide and can form homodimers. Tandem-repeat galectins have two linked recognition domains, whereas chimera-type Gal-3 has a recognition domain and an N-terminal region involved in multivalent organization.

Intracellular and extracellular galectin activities must be distinguished. Extracellular galectins can bind accessible cell-surface glycoconjugates; intracellular pools participate in processes that need not involve luminal endothelial glycans (32, 33). In glycan microarrays and cell-binding experiments, Stowell et al. (34) found differential recognition of extended LacNAc-containing and sialylated structures by Gal-1, Gal-2, and Gal-3. This is direct *in vitro* evidence that ligand chemistry matters, but it is not a study of aged brain endothelium.

The strongest candidate determinants of altered binding are the abundance and extension of LacNAc-bearing glycans, branching and multivalent presentation, and terminal modifications. Changes in alpha2,3- versus alpha2,6-sialylation can affect galectins differently; desialylation cannot be assumed to increase both Gal-1 and Gal-3 binding equally (34). For the aging study reviewed above, the measured bulk sialylation signals were unchanged, so age-related desialylation is not an established initiating event.

An architectural hypothesis is separate from a compositional hypothesis. Reduced mucin-domain coverage could change access to existing N-glycan or glycolipid ligands, but ligand loss could offset reduced steric shielding. Galectin retention also depends on spatial density, so a thinner layer could yield increased, decreased, or redistributed binding. Shedding may remove endothelial binding sites or generate soluble glycoproteins that compete for galectin; neither direction is established in aging-associated epilepsy. A vascular inflammation study demonstrated ADAM15-dependent glycocalyx shedding in its own experimental context (35), which does not supply a brain endothelial shedding rate during aging or seizures.

Accordingly, thickness alone, C1GALT1 transcript abundance, or a generic shedding marker cannot identify the relevant functional ligand. Testing the hypothesis requires paired structural measurements and direct Gal-1/Gal-3 binding, together with ligand identification and glycan-dependent competition controls. These distinctions also allow a null or reduced-binding result to falsify the proposed exposure mechanism rather than be reinterpreted as support.

## 7 Galectin and inflammasome experiments relevant to seizures

### 7.1 Galectin 3 in defined microglial systems

Sun et al. used male Sprague–Dawley rats aged 8–10 weeks in a lithium-pilocarpine model. TD139, 8 mg/kg/day intraperitoneally for 10 days beginning 4 hours after seizures, reduced behavioral seizure measures and hippocampal inflammatory markers. Seizures were video-monitored continuously for 2 weeks; prolonged EEG follow-up and persistence after treatment withdrawal were not established. In parallel, BV2 mouse microglia and neonatal rat primary microglia were exposed to 100 micromolar kainate for 24 h. TD139 or lentiviral Lgals3 knockdown reduced Iba1 and NLRP3/pyroptosis-associated readouts (9).

The measured proteins included NLRP3, ASC, cleaved caspase-1, and the N-terminal gasdermin-D fragment. Reduced readouts following pharmacological and genetic perturbation support Gal-3-dependent regulation in these systems. They do not establish direct Gal-3-NLRP3 binding, endothelial ligand engagement, or an aging-specific pathway. Cell-line and neonatal cultures also differ from aged adult microglia. Figure 1 therefore depicts the intervention and measured outcomes without molecular recruitment or oligomerization arrows (9).

**Figure 1:**
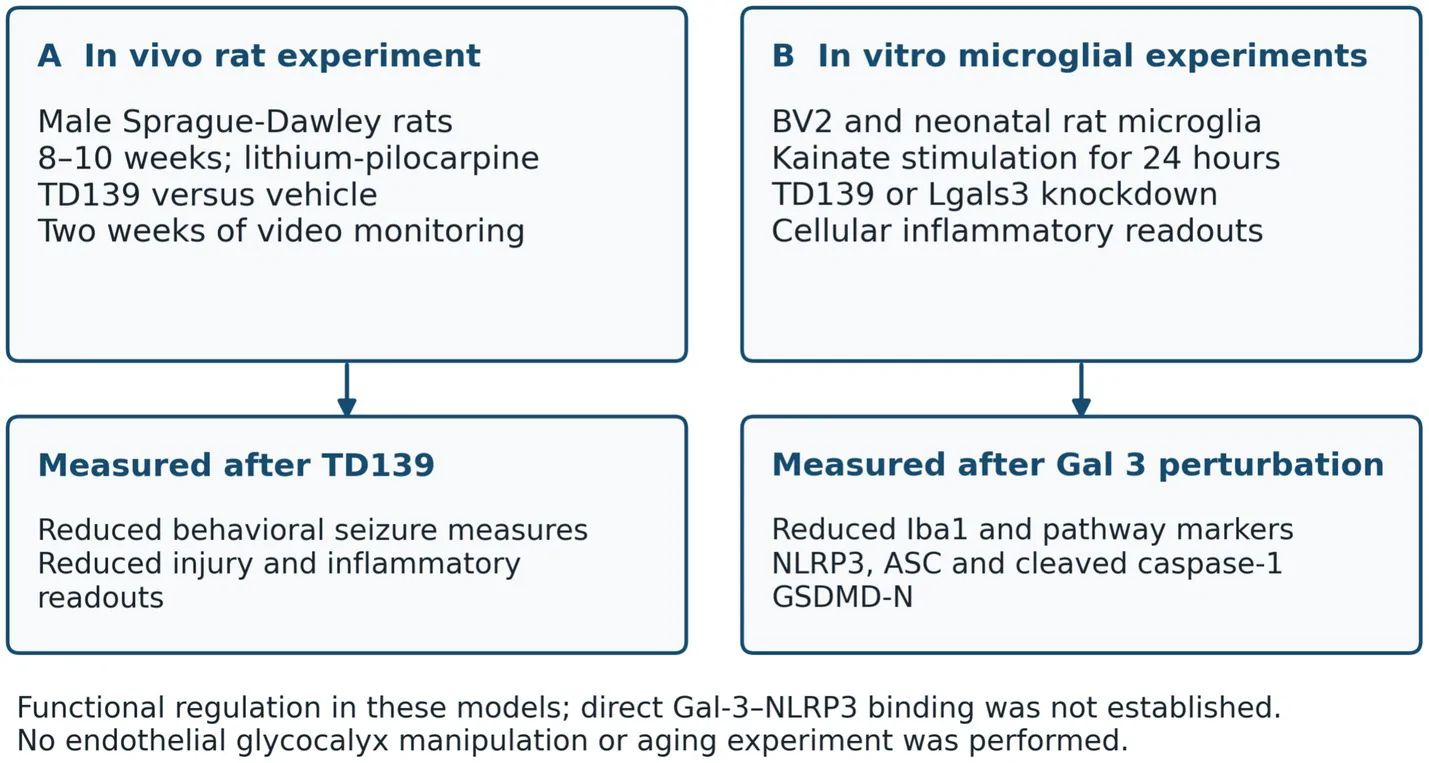
Experimental Gal-3 perturbations and their measured outcomes. **(A)** In vivo rat experiments and **(B)** cultured-microglial experiments from Sun et al. (9) are shown separately. Arrows connect interventions to measured changes, not molecular binding or inflammasome assembly. These experiments do not establish endothelial glycan mediation, an aging-specific pathway, or durable modification of epilepsy after treatment withdrawal.

NLRP3 is an intracellular inflammasome sensor; ASC is an adaptor, caspase-1 processes inflammatory substrates, and gasdermin-D cleavage is associated with pore formation. These intracellular readouts must not be drawn as if secreted Gal-3 at an endothelial surface physically recruits NLRP3. Mechanisms involving cytosolic galectin recognition of glycans exposed by damaged intracellular membranes represent a different compartment and would require direct evidence of those conditions in the seizure system. Such damage and direct binding were not established by the cited Gal-3 seizure experiment.

Burguillos et al. (36) investigated secreted Gal-3 and TLR4 in microglial systems, supporting an extracellular route to sustained microglial activation. This supplies a defined receptor-related precedent for microglial inflammation. It does not demonstrate a corresponding TLR4 mechanism on the luminal brain endothelial surface during aging or epilepsy.

### 7.2 Astrocytic inflammasome experiments and adenosine metabolism

Zhang et al. (37) used kainate-induced status epilepticus in mice and kainate-treated astrocytes. NLRP3 inhibition with MCC950, caspase-1 inhibition with Z-YVAD-FMK, and cellular knockdown experiments reduced inflammatory pathway readouts; increased astrocytic adenosine kinase (ADK) was linked to CREB, REST, and SP1 regulation. ADK metabolizes adenosine, an endogenous modulator of seizure susceptibility (38). These experiments identify an astrocytic pathway under the tested conditions. They do not show that endothelial glycan remodeling initiates it, and their ADK results should not be cited as direct evidence of impaired glutamate transport.

Aging-related microglial state changes provide another independent context. Single-cell RNA sequencing across the mouse lifespan identified heterogeneous microglial states rather than a uniform inflammatory phenotype (39); the concept of age-related priming describes altered responses to subsequent challenges (40). Neither observation demonstrates that endothelial Gal-3 binding causes age-dependent seizure susceptibility. Microglial Gal-3, astrocytic inflammasomes, and endothelial glycosylation therefore remain separate experimental components until tested together.

## 8 Context dependent galectin 1 findings

Gal-1 cannot be treated as a universally protective counterpart to Gal-3. Pacheco et al. administered exogenous Gal-1 to Wistar rats in an intrahippocampal pilocarpine status epilepticus model. Selected behavioral seizure classes were reduced, and inflammatory and neurodegenerative measures improved at 24 h and 15 days (41). These findings support benefit in the tested treatment context, but they do not establish prevention of chronic epilepsy or a protective endothelial glycan mechanism.

Starossom et al. (42) studied recombinant Gal-1 in activated microglial cultures and experimental autoimmune encephalomyelitis. Gal-1 engaged glycosylated CD45 and reduced inflammatory signaling, with *in vivo* effects on microglial activation and neurodegeneration. This provides mechanistic *in vitro* and *in vivo* evidence for immunoregulation in a non-epilepsy setting. Perillo et al. (43) demonstrated Gal-1-dependent T-cell apoptosis in another experimental system; this cannot be equated with reduced lymphocyte entry into the aging epileptic brain.

Conversely, Bischoff et al. found that deletion of endogenous Lgals1 markedly reduced pilocarpine-associated neuronal death in adult mice (44). The result cautions against extrapolating exogenous Gal-1 treatment effects to all endogenous Gal-1 functions. Cell source, extracellular versus intracellular location, concentration, and timing may account for different actions but require comparative testing. Figure 2 therefore compares defined studies and includes the opposing result. No experimentally validated Gal-1/Gal-3 ratio has been shown to determine epileptogenesis.

**Figure 2:**
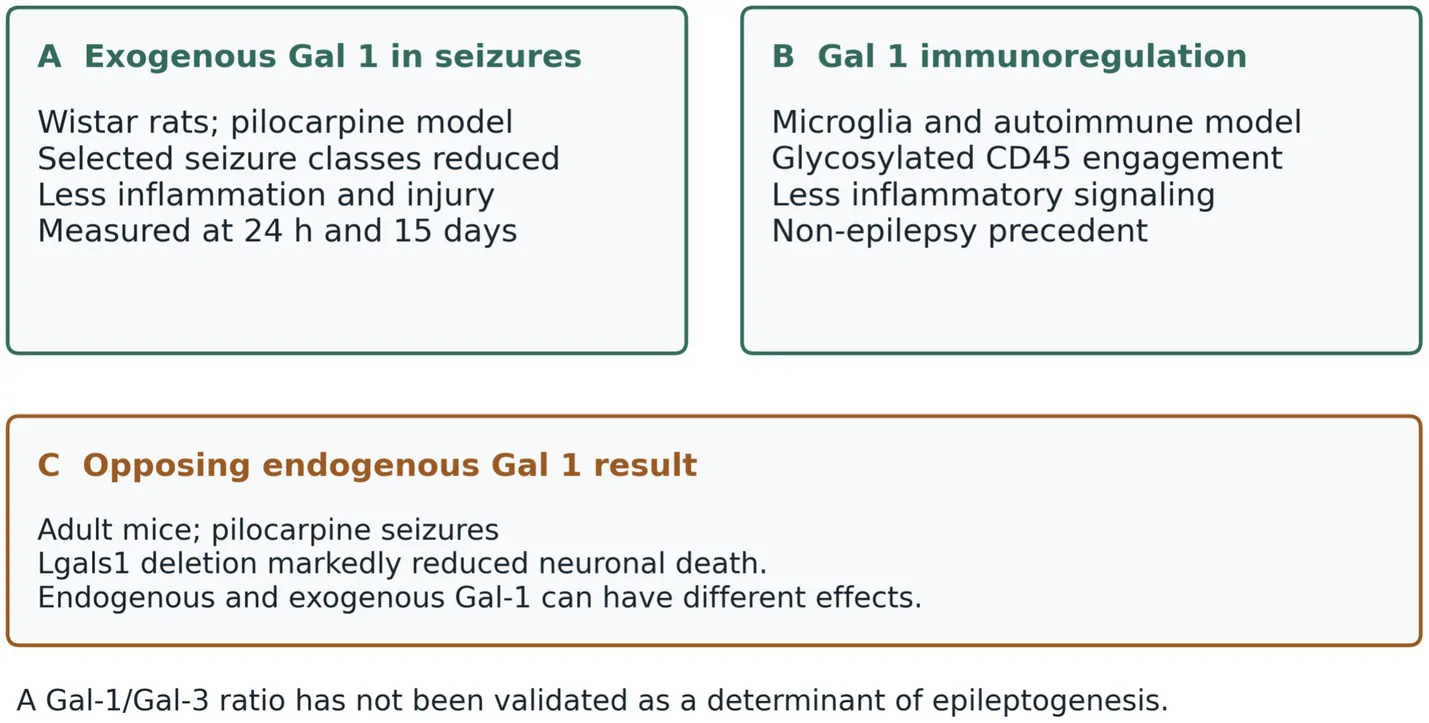
Context-dependent Gal-1 findings. **(A)** Exogenous Gal-1 in a Wistar-rat seizure model (41), **(B)** microglial immunoregulation in a non-epilepsy setting (42), and **(C)** protection associated with endogenous Lgals1 deletion in a mouse seizure model (44) are distinct observations. The schematic makes no claim that a Gal-1/Gal-3 balance determines epileptogenesis.

## 9 The convergence hypothesis and its anatomical requirements

### 9.1 Where could endothelial glycans and microglial galectin meet

The main anatomical limitation is that parenchymal microglia are outside the vascular lumen, whereas the glycocalyx emphasized here faces circulating blood. Endothelial cells, basement membranes, and surrounding vascular structures separate these compartments. Microglial Gal-3 upregulation therefore does not establish exposure of luminal endothelial glycans to microglia-derived Gal-3. BBB leakage to a tracer or albumin also does not demonstrate transport of Gal-3 in the opposite direction or access to a particular endothelial surface.

Three routes can be formulated as alternatives to test. First, blood-derived Gal-3 could encounter luminal glycans; its source and concentration would have to be measured. Second, microglia-derived extracellular Gal-3 could act on an abluminal endothelial surface, requiring separate mapping of those glycans rather than assuming that they match the luminal glycocalyx. Third, altered barrier transport or major injury could permit intercompartmental exposure, but that route requires tracer, localization, and transport evidence. None is established here as the operative pathway in aged epilepsy.

Microglia could also influence endothelium indirectly through cytokines, without Gal-3 ever binding the luminal glycocalyx. Conversely, glycosylation restoration could reduce leakage of blood proteins and consequently reduce glial inflammation through a galectin-independent mechanism. The convergence hypothesis must be compared with these explanations, rather than treating any concurrent BBB and inflammatory improvement as proof of the proposed coupling.

### 9.2 Proposed sequence and competing explanations

The model predicts that a defined endothelial glycan alteration precedes or accompanies a measurable change in galectin engagement in an identified compartment, followed by Gal-3-dependent vascular or inflammatory changes that influence persistent seizure outcomes. Each transition is untested in the complete aging-associated epilepsy pathway. Figure 3 separates supported experimental components from these proposed connections.

**Figure 3:**
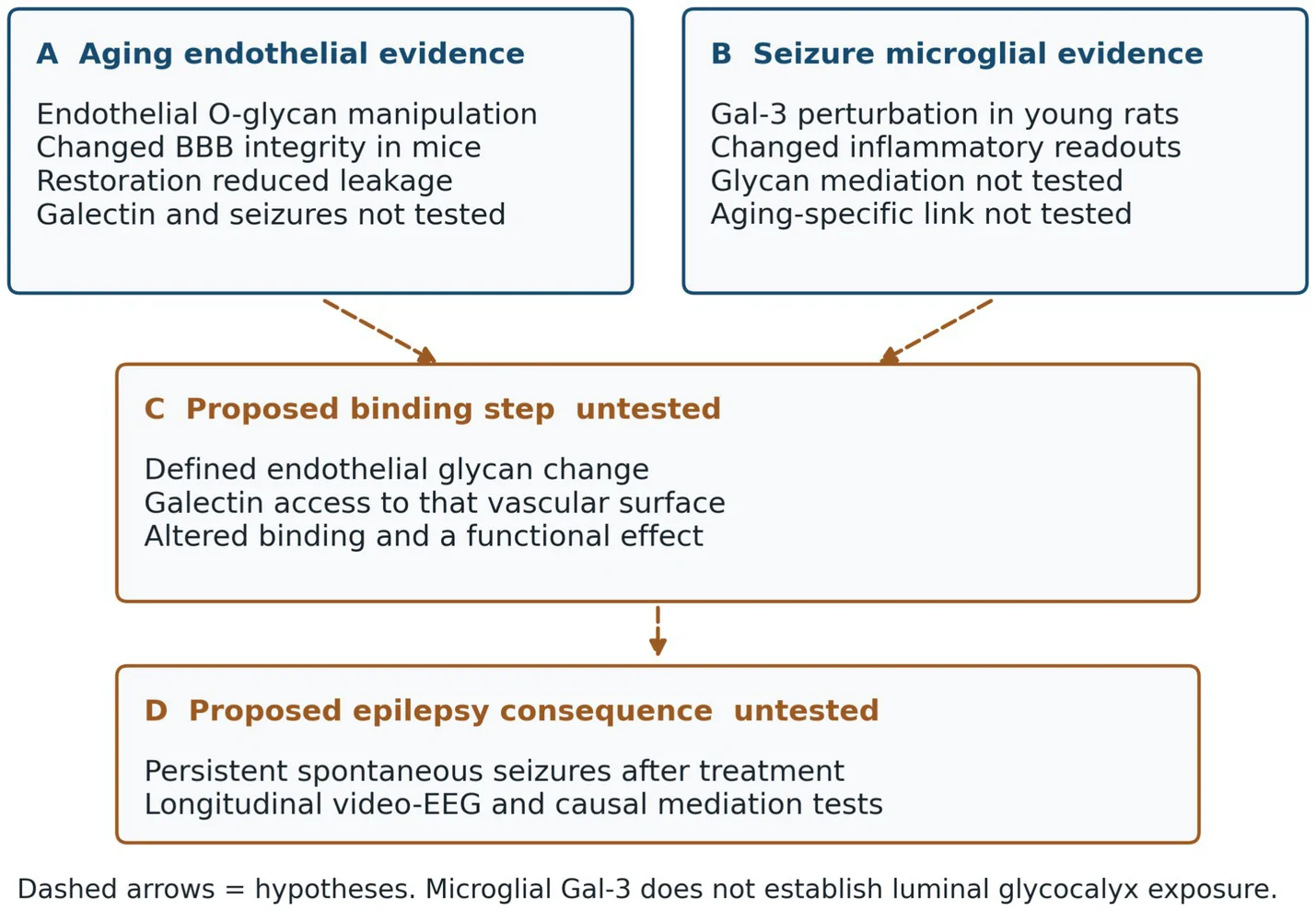
Convergence hypothesis with explicit evidence boundaries. Panels **(A)** and **(B)** summarize model-specific experimental components (8, 9). Dashed connections to panels **(C)** and **(D)** are untested: the glycan ligand, galectin source, vascular surface, binding change, and persistent seizure consequences must be established in the same system. The graphic does not assume that parenchymal microglial Gal-3 reaches luminal glycans.

Direct immune and neuronal galectin actions remain competing explanations. A Gal-3 intervention could reduce microglial inflammation independently of endothelial glycosylation; a Gal-1 intervention could affect immune cells or neurons without any glycocalyx contribution. Aging, BBB leakage, galectin changes, and seizures could also share an upstream cause. A feed-forward circuit is possible, but its direction, timing, and necessity must be established with independent perturbations and mediation experiments.

### 9.3 Candidate downstream routes to neuronal excitability

If altered endothelial glycans change galectin engagement at an accessible vascular surface, the next required step is a functional endothelial response. Candidate effects include altered receptor organization, inflammatory signaling, or selective transport. None has been demonstrated as the consequence of aging-dependent endothelial Gal-3 binding in an epilepsy model. The hypothesis therefore requires measurements linking binding to endothelial function, rather than assuming that more galectin fluorescence means a more permeable BBB.

One possible downstream route is vascular leakage followed by astrocytic dysfunction. If the putative binding event increases albumin entry, astrocytic TGF-beta signaling and impaired Kir4.1-dependent potassium buffering provide a previously demonstrated mechanism through which vascular injury can promote epileptiform activity (18). Increased extracellular potassium could facilitate neuronal depolarization and network recruitment. The albumin–astrocyte link is supported experimentally; its initiation by endothelial galectin binding is proposed. A change in transcytosis without sufficient albumin entry would not establish this route.

A second possibility is endothelial signaling to glia followed by inflammatory changes in the neuronal environment. Microglial Gal-3/TLR4 signaling and astrocytic NLRP3-associated ADK regulation supply separate cellular precedents (36, 37). Increased ADK could reduce the availability of anticonvulsant adenosine (38). These observations do not establish a serial endothelial–microglial–astrocytic pathway, and direct glial galectin actions remain an alternative explanation. Connecting either candidate route to epileptogenesis would require persistent spontaneous seizures, together with evidence that blocking the intervening astrocytic or inflammatory response prevents the effect of endothelial glycan perturbation.

### 9.4 Seizures as a potential cause of vascular remodeling

The relationship may also run from seizures to the vascular surface. Experimental epileptiform activity induces endothelial adhesion factors, and interference with leukocyte–endothelial adhesion modifies seizure-related pathology (16, 17). These findings establish that vascular responses can accompany seizure activity; they do not show that seizures alter endothelial O-glycan chemistry or luminal Gal-3 binding. In a separate vascular inflammation model, ADAM15-dependent glycocalyx shedding contributed to leakage (35). This provides a precedent for inflammation-driven surface remodeling, but its occurrence in the aging epileptic brain remains to be tested.

A proposed feed-forward circuit would therefore begin with seizures or inflammatory vascular dysfunction, followed by altered glycan synthesis, turnover, or shedding; changed galectin engagement could then sustain vascular or glial responses and increase subsequent seizure susceptibility. The identity and direction of the glycan change cannot be assumed. Ligand loss or compensatory repair could weaken rather than amplify signaling. Serial measurements before seizure induction, after the acute insult, and during chronic epilepsy are needed to distinguish antecedent vulnerability from a consequence of seizures and to determine whether the proposed feedback persists after acute inflammation subsides.

## 10 Therapeutic and biomarker implications

### 10.1 Experimental interventions and their limits

Restoring endothelial O-glycosylation is experimentally feasible in aged mice, and inhibiting Gal-3 modifies outcomes in a separate young-rat seizure model (8, 9). These observations do not demonstrate that one intervention acts through the other. Table 1 separates the relevant settings and endpoints, including peripheral vascular interventions that offer translational precedent only.

**Table 1:** Intervention evidence and limits for aging-associated epilepsy.

Intervention	Experimental setting	Measured findings	Inference limit
Endothelial O-glycan restoration (8)	Male aged C57BL/6 mice; endothelial AAV-C1GALT1 or AAV-B3GNT3	Improved mucin labeling and BBB tracer retention; B3GNT3-specific significant cognitive benefit.	Brain aging proof of concept; no galectin-binding or seizure experiment.
Glycocalyx repair (45)	Experimental sepsis; glycocalyx restoration intervention	Vascular glycocalyx and permeability outcomes.	Peripheral inflammatory model; no proof of brain glycocalyx–galectin mediation.
Sulodexide (46)	Rat carotid balloon-injury model	Improved endothelial/glycocalyx measures after vascular injury.	Large-artery repair is not a BBB or epilepsy experiment.
Sulodexide (47)	Patients with type 2 diabetes	Peripheral glycocalyx and vascular permeability measurements.	Human peripheral evidence; CNS and seizure effects not established.
Gal-3 inhibition (9)	Young male rats; lithium-pilocarpine; TD139 versus vehicle	Reduced behavioral seizure measures, injury and inflammatory readouts.	Two-week video follow-up; durable EEG-confirmed epilepsy modification unresolved.
Exogenous Gal-1 (41)	Wistar rats; intrahippocampal pilocarpine	Selected seizure classes and tissue inflammation/injury improved.	Context-specific benefit; endogenous Gal-1 can have different effects.
Inhaled TD139/GB0139 (48)	Human idiopathic pulmonary fibrosis study	Pulmonary target engagement and formulation-specific clinical information.	Does not establish therapeutic CNS exposure or epilepsy efficacy.

AAV approaches require assessment of endothelial targeting, expression level, immunogenicity, durability, and safety. Systemic glycocalyx interventions studied in sepsis, carotid injury, or diabetes do not establish brain endothelial repair in epilepsy (45–47). Inhaled TD139/GB0139 showed pulmonary target engagement in an idiopathic pulmonary fibrosis study (48); this does not establish therapeutic CNS exposure, neurological safety, or epilepsy efficacy. Figure 4 presents the translational questions without a clinical seizure-protection claim.

**Figure 4:**
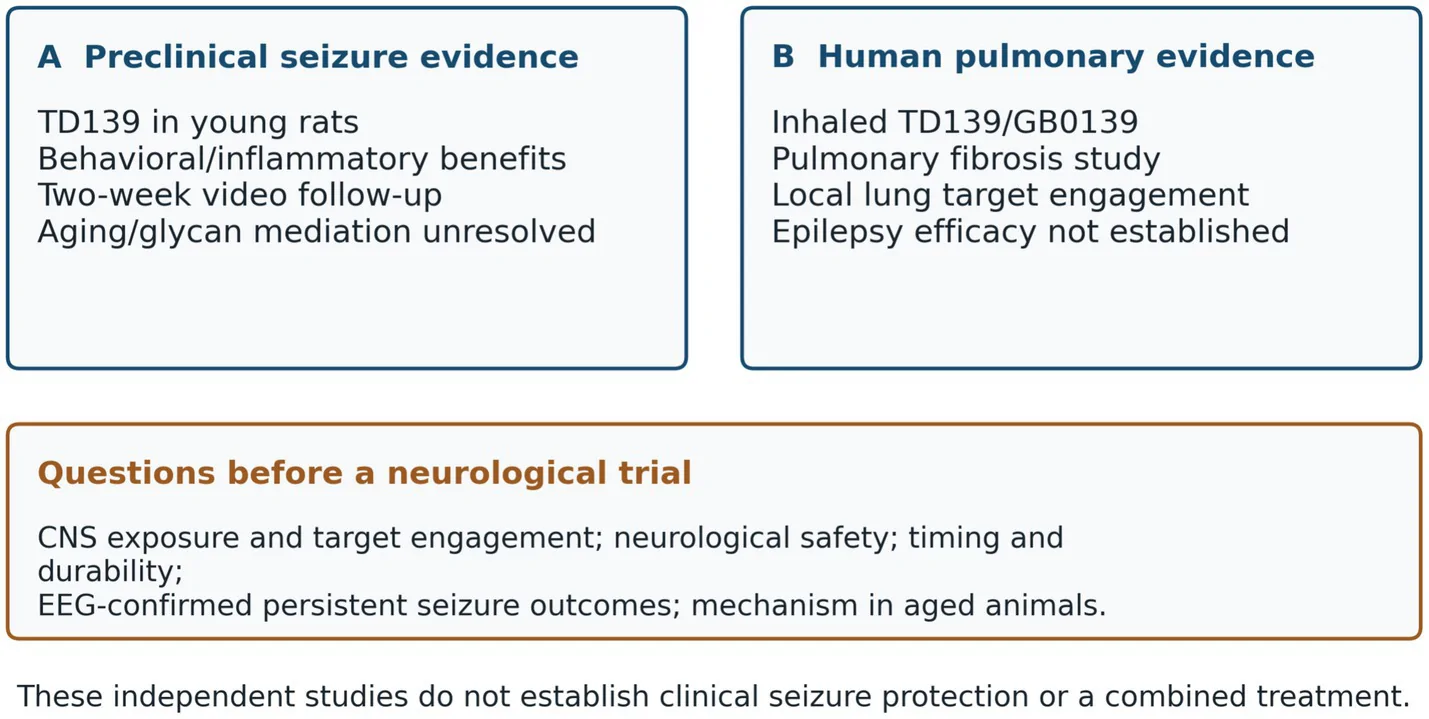
Preclinical rationale and neurological translation gaps. **(A)** Experimental seizure findings in young rats (9) and **(B)** pulmonary target engagement in humans (48) are shown as independent evidence streams. Neither demonstrates clinical epilepsy efficacy or the proposed aging endothelial mechanism.

Combination treatment should be considered only as an experimental design. A factorial comparison of endothelial glycosylation restoration and cell-specific Gal-3 perturbation can test whether their effects interact. Improvement in BBB leakage, inflammatory markers, or acute seizure severity must be separated from a durable reduction in spontaneous recurrent seizures after treatment has ended.

### 10.2 Biomarker specificity and timing

Circulating Gal-3 and peripheral glycocalyx markers cannot be assumed to identify the proposed brain endothelial mechanism. Blood biomarker studies in epilepsy highlight the need to distinguish seizure-related injury from diagnostic or prognostic information (49). Syndecan or glycosaminoglycan fragments can originate from other vascular beds, while circulating Gal-3 can reflect processes outside the CNS (6, 33). Multimorbidity in older adults adds further ambiguity.

Longitudinal panels could pair BBB imaging with inflammatory, glial, neuroaxonal, and glycan measurements. Neurofilament light and GFAP have been studied in seizures and epilepsy, but their relevance to this specific mechanism remains unresolved (50). Pre-seizure, postictal, and interictal sampling would be necessary to distinguish antecedent vulnerability from a response to seizures. The review does not claim that Gal-3 or glycocalyx markers currently predict recurrence, cognitive decline, or response to glycosylation therapy.

## 11 Experiments needed to evaluate the hypothesis

### 11.1 A compartment resolved *in vitro* test

The first experiment should manipulate endothelial glycosylation independently of galectin abundance. Brain endothelial cultures under flow, with appropriate pericyte and astrocyte support, could compare C1GALT1 or B3GNT3 perturbation with control and rescue conditions. Luminal and abluminal Gal-3 exposure should be varied independently, with microglial conditioned medium tested in a separate arm. BBB-on-chip work provides a platform precedent (51); a chip containing all relevant cells and an aging phenotype is a proposed extension, not an already validated model of this mechanism.

Measurements should include defined glycan structures, mucin labeling, surface ligand density, direct galectin binding, permeability, and endothelial viability. Lactose competition, a suitable carbohydrate-binding-defective galectin control, endotoxin testing, and ligand-specific perturbation would help distinguish carbohydrate-dependent binding from nonspecific injury. Transcript measurements alone cannot substitute for glycomic or glycoproteomic characterization.

To resolve directionality, conditioned medium from microglia with and without Lgals3 perturbation should be compared under matched inflammatory conditions. A change in endothelial function that persists despite blocking galectin carbohydrate recognition would argue against the proposed glycan-binding step. A change caused only by cytokine exposure would support an indirect inflammatory route. Positive binding should also be connected to a functional outcome; altered fluorescence alone would not establish the mechanism.

### 11.2 An aging and epilepsy factorial experiment

Young-adult and aged animals should be studied with both sexes, age-matched non-seizure controls, and a specified seizure-induction protocol. Endothelial glycosylation and microglial versus endothelial Gal-3 should be perturbed separately and in combination. The vascular compartment, ligand chemistry, and cellular source of Gal-3 must be measured rather than inferred from hippocampal homogenates. Continuous video-EEG should assess acute seizures and persistent spontaneous recurrent seizures over a suitable long-term interval, including after intervention withdrawal.

The hypothesis would gain support if endothelial glycan changes precede altered galectin binding, correcting those glycans reverses the binding change, and seizure-related effects depend on galectin recognition in the same compartment. If restoration improves leakage without changing galectin engagement, the barrier benefit remains valuable but the proposed mediation is unsupported. If Gal-3 perturbation is equally effective across endothelial glycan states, an independent microglial pathway is a stronger explanation. Negative results must be interpreted only after confirming target engagement and adequate measurement sensitivity.

To test the downstream and reverse routes, longitudinal cohorts should pair video-EEG with measurements of endothelial glycans, compartment-specific galectin binding, transport, and glial responses. Regional sampling should include the hippocampus and cortex rather than presume a uniform aging phenotype (22, 23). Separate cohorts sampled before induction, shortly after seizures, and during the chronic phase would establish temporal order. Perturbing the endothelial binding step and a candidate downstream mediator independently would test necessity. Comparing groups with matched initial seizure burden would help distinguish a direct vascular effect from remodeling secondary to fewer seizures. Glycan changes appearing only after seizures would weaken an initiating role while remaining compatible with a maintenance loop.

### 11.3 Sex and etiology

The male-only aging glycocalyx experiments and female-only Elahy aging comparison cannot establish a sex interaction by comparison across studies. Peripheral measurements in older adults suggest that glycocalyx phenotypes can differ by sex (52), but this is not direct evidence for sex-specific cerebral galectin coupling. Both sexes should therefore be included and analyzed prospectively.

Post-stroke, Alzheimer-associated, traumatic, and unexplained late-onset epilepsies should also be evaluated separately. They differ in vascular injury, inflammation, and network remodeling (11, 13). A mechanism demonstrated after severe vascular injury might not operate in physiological aging with a relatively intact barrier. Human confirmation would require compartment-relevant measurements linked to clinical seizure phenotypes and confounder-aware longitudinal analysis.

## 12 Discussion and conclusion

The strongest available evidence establishes adjacent components: endothelial O-glycosylation contributes to BBB integrity in aged mice, vascular leakage can alter astrocytic homeostasis, and Gal-3 perturbation changes inflammatory and seizure-related outcomes in selected experimental systems. None of these studies demonstrates the complete sequence from aging endothelial glycan remodeling to compartment-specific Gal-3 engagement and chronic epileptogenesis.

The central hypothesis is therefore conditional. Endothelial remodeling could influence galectin-dependent signaling if it changes relevant ligands and if the galectin reaches the affected surface. Ligand loss, reduced binding, indirect cytokine effects, and galectin-independent BBB repair are plausible alternatives. Gal-1 findings further show why galectins should not be assigned fixed beneficial or detrimental roles across models. Regional and transport-specific BBB aging further limits a uniform exposure model. The proposed mechanism may contribute to seizure vulnerability, follow seizure-associated vascular inflammation, or participate in a bidirectional circuit; these possibilities require temporal and mediation tests.

The contribution of this review is to define the missing causal tests and the findings that would argue against the mechanism. Therapeutic or biomarker claims specific to aging-associated epilepsy should depend on those tests. Until they are completed, the glycocalyx-galectin connection remains a convergence hypothesis rather than an established explanation for late-onset epilepsy.
